# Clinical nurses’self-assessed knowledge, beliefs, and practice in nutritional management of chronic disease patients: A cross-sectional survey in Zhejiang Province

**DOI:** 10.1097/MD.0000000000049154

**Published:** 2026-06-05

**Authors:** Shuyan Li, Aixia Wang, Xiaoxia Huang, Huadi Yuan, Haiyan Zhou, Qiaoling Zhang, Yuehua Dai, Jiaying Tang, Xiuqin Feng

**Affiliations:** a Department of Nursing, The Second Affiliated Hospital, Zhejiang University School of Medicine, Hangzhou, China; b Department of Nursing, The Second Affiliated Hospital of Wenzhou Medical University, Wenzhou, China; c Community Health Service Center of Nanxing Subdistrict, Shangcheng District, Hangzhou, China.

**Keywords:** beliefs, chronic disease patients, nurses, nutritional management, practice, self-assessed knowledge

## Abstract

Malnutrition among hospitalized chronic disease patients is a global concern, with studies showing that nearly 30 to 50% of chronic disease patients experience disease-related malnutrition (DRM) during hospitalization. DRM can significantly affect patients’recovery, increase the length of hospital stay, and lead to higher mortality rates. To assess the current state of nutritional management implementation among clinical nurses in Zhejiang Province and determine the factors that influence their self-assessed knowledge, beliefs, and practices concerning nutritional support for chronic disease patients. A cross-sectional study was conducted from October 31, 2024 to December 31, 2024, involving 1561 nurses from 40 hospitals in Zhejiang Province. A questionnaire was distributed via a quick response code, covering general information of respondents and hospitals, nutritional management models, and nutritional support self-assessed knowledge, beliefs, and practice. Descriptive statistics, χ^2^ tests, t-tests, analysis of variance, rank sum tests, and regression analyses were performed using SPSS 27.0. Most departments had established nutritional management procedures (69.63%), systems (67.01%), and training plans (69.25%) for chronic disease patients, and 80.85% had formed multidisciplinary nutrition support teams. However, only 57.98% conducted regular follow-ups on nutritional status after discharge. Factors influencing self-assessed knowledge of nutritional support included age, job title, being a nutrition support specialist nurse, and the presence of nutritional management systems. Beliefs about nutritional support were mainly affected by regular assessment of nutritional management, emergency plans for adverse events, and post-discharge follow-up. The implementation of nutritional support was influenced by regular assessment, protocols for handling enteral nutrition complications, and post-discharge follow-up. Most hospital departments have made progress in the foundational aspects of nutritional management for chronic diseases but improvements are needed in practice and management. Our results provide a basis for enhancing nurses’ participation and effectiveness in chronic disease nutrition management.

## 1. Introduction

Despite major strides in global healthcare, disease-related malnutrition (DRM) remains a persistent and growing concern among hospitalized patients, particularly in patients with chronic diseases and critical illnesses.^[1]^ DRM has substantial clinical and functional consequences: it is associated with prolonged length of hospital stay, higher rates of infection and other complications, impaired wound healing, accelerated muscle wasting and sarcopenia, increased readmission rates, poorer functional recovery, and increased mortality.^[2]^ These adverse outcomes impose additional burdens both on patients and health systems and underscore the importance of timely screening and effective nutritional management.^[2]^ In the United States, the 8 leading causes of death include cardiovascular disease, ischemic stroke, type 2 diabetes, fatty liver disease and cirrhosis, cancer, osteoarthritis, and childhood and adult obesity, and all of these are associated with DRM.^[3]^ The incidence of DRM ranges between 20% and 50% depending on the study population, diagnostic method, and hospital environment.^[3]^ In Europe, the prevalence is 20 to 30%, with rates as high as 32 to 58% among elderly patients and those with malignant diseases. The incidence is 27 to 39%^[4]^ throughout Asia and 30 to 60% in China, being particularly high in cancer patients (47.7–49.0%).^[5]^ DRM can prolong hospital stays, increase the incidence of complications and readmission rates, and even increase the risk of patient mortality.^[6]^

Nutritional support is an indispensable part of therapy in clinical practice, improving patient prognosis and clinical outcomes. Currently, the main methods adopted clinically include enteral, parenteral, and combined support. Enteral support not only maintains the integrity of the patient’s gastrointestinal mucosal structure and function but also enhances immunity, preventing bacterial translocation and thus reducing the incidence of infections.^[7]^ When patients cannot receive any or enough enteral nutrition, parenteral nutrition needs to be provided.^[8]^ Screening and assessing the nutritional status of hospitalized patients can identify those at nutritional risk or who are malnourished early.^[9]^ Implementing early interventions for such patients can improve their nutritional status, shorten hospital stays, reduce medical expenses, lower the incidence of complications, and even decrease mortality rates.^[10]^

However, the effective implementation of standardized nutritional protocols relies on professional nursing care, and the quality of nursing directly affects patient prognosis and outcome.^[11]^ During nutritional management, nurses play a vital role by administering nutritional infusions, maintaining feeding tubes, and preventing or managing complications. Previous studies have identified several gaps in nursing-related nutritional care: many nurses lack formal education in clinical nutrition, adherence to enteral nutrition protocols is inconsistent, routine nutritional screening and documentation are not universally performed, and post-discharge nutritional follow-up is often inadequate. These gaps in knowledge, attitudes, and practice^[12]^among nurses may directly limit the effectiveness of hospital nutritional programs and the capacity to prevent or treat DRM. The safe and effective implementation of nutritional support by nurses, both in domestic and international settings, still requires improvement. A survey on nutritional knowledge among intensive care unit nurses revealed that most had not received formal nutritional education, and some did not consistently adhere to enteral nutrition protocols.^[13]^ Nearly all participants acknowledged having knowledge gaps in nutritional care.

Cultural and social factors influence patients’ dietary behaviors during hospitalization and may interact with DRM risk. Food preferences, traditional dietary restrictions, language barriers, and the acceptability of hospital meals or oral nutritional supplements can affect intake and adherence to prescribed nutrition plans. Awareness of and adjustments for patients’ social and cultural backgrounds are therefore important components of individualized nutritional management, and nursing assessments that capture these aspects may facilitate more acceptable and effective interventions.

To clarify how these elements interrelate, we propose a conceptual framework that integrates nurse-level factors (knowledge, beliefs/attitudes, and practices), patient factors (type of chronic disease, nutritional risk, social/cultural background, dietary pattern), and organizational factors (existence of protocols, multidisciplinary nutrition support teams, training programs, and discharge follow-up). In this model, improved nurse knowledge and positive beliefs (attitudes) enhance practice behaviors (e.g., routine screening, timely initiation of enteral/parenteral support, complication management, and discharge follow-up), which in turn lead to better nutritional management and reduced DRM.

To guide our investigation, we developed the Integrated Nurse-Patient-Organization (NPO) Framework for Nutritional Management. This framework outlines 3 interconnected determinants of effective nutritional care for chronic disease patients. At the nurse level, individual competency, encompassing knowledge, beliefs, and reported practices, forms the immediate basis for care delivery. The patient level introduces specific needs shaped by disease type, nutritional risk, and socio-cultural context, necessitating individualized approaches. The organizational level provides the structural foundation through protocols, multidisciplinary teams, training, and follow-up systems. These levels interact dynamically. Supportive organizational structures enable nurses to apply their knowledge effectively, while patient factors require nurses to adapt standardized protocols. Ultimately, the alignment of competent practice, responsive systems, and patient-centered adaptation is essential for improving nutritional outcomes. This NPO Framework informed our study design and variable selection.

This study aims to assess the current implementation status of nutritional management among clinical nurses in Zhejiang Province and to determine factors that influence nurses’ self-assessed knowledge, beliefs, and practices concerning nutritional support for chronic disease patients. Our primary research questions are: What are the current levels of self-assessed knowledge, beliefs, and practice regarding nutritional management among clinical nurses in Zhejiang? Which nurse, patient, and organizational factors are associated with higher self-assessed knowledge, more positive beliefs, and better practice? The Nursing Nutrition Professional Committee of the Zhejiang Nutrition Society plays a central role in advancing nutritional care across the province through standard setting, professional training, public education, and research coordination. Leveraging this platform, we investigated how clinical nurses in Zhejiang currently manage patient nutrition.

## 2. Methods

### 2.1. Study participants

**Figure 1.**
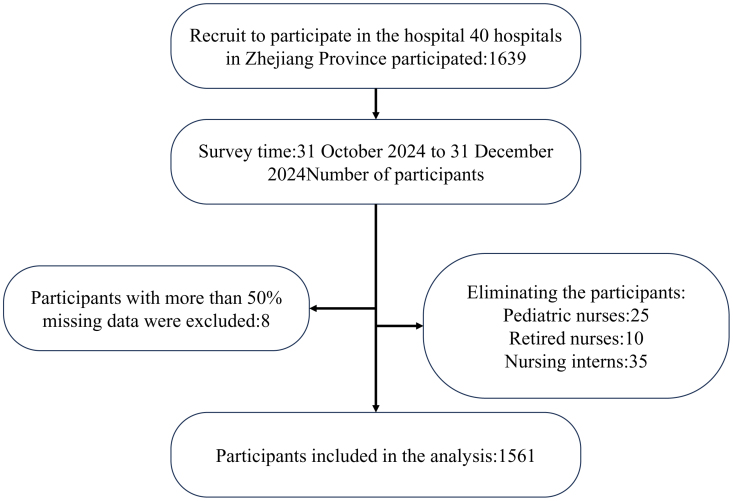
Flow chart of the study. We sent the questionnaire to the WeChat and 1639 participants responded, out of which 78 respondents were excluded.

This study adopted a cross-sectional survey design to assess the self-assessed knowledge, beliefs, and practices of clinical nurses regarding nutritional support for chronic disease patients. The study used a convenience sampling technique to select participating hospitals and nurses.In total, 40 hospitals in Zhejiang Province took part in this study. A quick response code (QRC) for the questionnaire was sent to a workgroup of the abovenamed committee. This group is made up of the heads of nursing departments and head nurses of nutrition-related departments in various hospitals across the province. They were asked to share the QRC with their colleagues from October 31, 2024 to December 31, 2024. Eligible participants were required to have experience in nutrition-related nursing. Pediatric nurses, retired nurses, and nursing interns were excluded. To maintain consistency with the study’s focus on nutritional practices in general ward settings, nurses working in intensive care units and oncology departments were not eligible for this study, as the survey was distributed only to nurses in nutrition-related departments. Ethical approval was obtained from the Ethics Committee of the Second Affiliated Hospital, School of Medicine, Zhejiang University (Hangzhou, Zhejiang Province, China, 20251038).Using G*Power 3.1, we calculated the required sample based on a conservative estimate of a small-to-medium effect size (Cohen’s f^2^ = 0.05), an alpha of 0.05, a power of 0.80, and accounting for up to 15 predictors in our planned regression models. This calculation indicated a minimum requirement of approximately 1200 participants. Our final sample of 1561 nurses comfortably exceeds this threshold. Participant inclusion process are presented in Figure 1.

### 2.2. Method of data collection

The questionnaire consisted of 6 sections: respondent demographics, including age, sex, education level, and years of work experience; institutional characteristics, such as hospital tier, location, and teaching status; departmental nutrition management models, covering current processes, policies, and emergency protocols; self-assessed self-assessed knowledge of nutritional support (gauged by the statement, “Compared to most people, I know a lot about nutrition”); beliefs related to nutritional support, including recognition of the importance of nutritional risk screening and assessment for patients with chronic diseases (Table S1); and nutrition support behaviors, including adherence to the “Group Standards for Enteral Nutrition Support in Adults”^[14]^and the “Group Standards for Placement and Maintenance of Nasoenteric Tubes in Adults”^[15]^issued by the Chinese Nursing Association in 2021 (Table S2).

The questionnaire on beliefs about nutritional support for patients with chronic disease was independently developed by the research team using the Delphi method. It comprised 10 items rated on a 5-point scale, with total scores ranging from 10 to 50. Higher scores indicated stronger beliefs. The scale demonstrated good internal consistency (Cronbach α = 0.856) and construct validity (Kaiser–Meyer–Olkin [KMO] value = 0.898).

The questionnaire assessing implementation of nutritional support practices was also independently developed using the Delphi method. It consisted of 18 items rated on a 5-point Likert scale, with total scores ranging from 18 to 90. Higher scores indicated poorer implementation. This instrument demonstrated excellent reliability (Cronbach α = 0.902) and sampling adequacy (KMO value = 0.928).

### 2.3. Data analysis

All statistical analyses were performed using SPSS version 27.0 (IBM Corp., Armonk). We used descriptive statistics to summarize participant characteristics (such as age, sex, education level, years of work experience, professional title, role, and nutrition support specialist status) and key outcome measures (self-assessed knowledge, beliefs, and implementation of nutritional support). For categorical variables, chi-square tests were used to examine associations with nutritional knowledge. Continuous variables with a normal distribution are expressed as mean ± standard deviation and were compared using independent-samples t-tests or analysis of variance (ANOVA). For non-normally distributed variables, medians with interquartile ranges are reported, with differences assessed using rank sum tests. Multiple regression analyses were conducted to further explore influencing factors. A *P*-value of <.05 was considered statistically significant for all tests, including chi-square tests for categorical data, t-tests and ANOVA for continuous variables, and regression analyses for identifying predictors of self-assessed knowledge, beliefs, and nutritional support practice.

## 3. Results

### 3.1. Selection of hospitals for investigation

Participants were recruited from 40 hospitals across Zhejiang Province (detailed institutional characteristics are given in Table 1). Tertiary class A hospitals accounted for the largest share (70.00%), followed by tertiary class B hospitals (22.50%), while secondary class A, secondary class B hospitals, and community health service centers each represented 2.50%. Geographically, respondents were primarily concentrated in Hangzhou (37.41%), Jinhua (12.68%), Jiaxing (10.63%), and Wenzhou (7.50%), with the remaining participants being distributed across other cities. Most participating hospitals (87.50%) were designated as teaching institutions. Additionally, 67.50% employed specialized nutrition support nurses, of whom 66.67% had clearly defined responsibilities for this role. Further institutional details are provided in Table 1.

**Table 1:** Hospitals’ characteristics (n = 40).

Variables	Category	Frequency	Composition ratio (%)
Hospital Grade	Level 3, Class A	28	70.00
	Level 3, Grade B	9	22.50
	Level 2, Grade A	1	2.50
	Level 2, Grade B	1	2.50
	Community Health Service Center	1	2.50
Region	Hangzhou	584	37.41
	Huzhou	63	4.04
	Jiaxing	166	10.63
	Jinhua	198	12.68
	Lishui	100	6.41
	Ningbo	99	6.34
	Quzhou	45	2.88
	Shaoxing	133	8.52
	Taizhou	39	2.50
	Wenzhou	117	7.50
	Zhoushan	17	1.09
Teaching Hospital	Yes	35	87.50
	No	5	12.50
Are there nutrition support specialist nurses available?	Yes	27	67.50
	No	6	15.00
	Unclear	7	17.50
If so, are there job responsibilities for nutrition support specialist nurses?	Yes	18	66.67
	No	9	33.33
If so, is there a specialized outpatient clinic for nutritional support nursing?	Yes	12	44.44
	No	15	55.56

### 3.2. Participants’ characteristics

According to the survey distribution records, a total of 1639 eligible nurses received the QR code link, and 1561 valid responses were collected. This yields a response rate of 95.3%. In all, 1561 nurses participated in the survey, with the majority being aged 20 to 40 years. The 20 to 30 and 31 to 40 age groups accounted for 40.23% and 40.49% of respondents, respectively. Educational attainment was high, with 87.64% holding a bachelor’s degree or above and 85.27% identified as having undergraduate degrees. Regarding work experience, 33.63% had been practicing for 11–20 years, reflecting a relatively experienced cohort. In terms of professional titles, nurse practitioners (39.21%) and supervisor nurses (36.77%) made up the largest proportions. Most respondents (92.12%) were clinical nurses, while only 13.97% were designated as nutrition support specialist nurses. Additional demographic characteristics are presented in Table 2.

**Table 2:** Participants’ characteristics (n = 1561).

Variables	Category	Frequency	Composition ratio (%)
Age (years)	20 to 30	628	40.23
	31–40	632	40.49
	41–50	266	17.04
	≥51	35	2.24
Education	Senior High School/Vocational High School	4	0.26
	Junior college	193	12.26
	Bachelor’s degree	1331	85.27
	Master’s degree	33	2.11
Work experience (years)	<1	76	4.87
	1–3	216	13.84
	4–6	215	13.77
	7–10	301	19.28
	11–20	525	33.63
	>20	228	14.61
Job titles	Nurse	230	14.73
	Registered Nurse	612	39.21
	Nurse Supervisor	574	36.77
	Deputy Chief Nurse	124	7.94
	Chief Nurse	21	1.35
Role	Nursing Administrator	123	7.88
	Clinical Nurse	1438	92.12
Nutrition support specialist nurses or not?	Yes	218	13.97
	No	1343	86.03

### 3.3. Current management status in the surveyed departments

The results revealed that 69.63% of departments had established nutritional management procedures for patients with chronic diseases, and 67.01% had implemented corresponding management systems. Additionally, 69.25% reported training programs that incorporated both theoretical self-assessed knowledge and practical skills in nutritional nursing. A collaborative care model was evident, with 80.85% of departments forming multidisciplinary nutrition support teams. Nutritional risk screening was widely practiced, with 95.77% of departments conducting screenings for patients with chronic diseases, half of which (50.48%) were jointly performed by doctors and nurses. Most departments (88.60%) had developed protocols to manage potential complications. However, only 57.98% reported conducting regular follow-ups or monitoring patients’ nutritional status after discharge. Additional departmental practices are detailed in Table 3.

**Table 3:** Characteristics of nutritional management of chronic diseases.

Variables	Category	Frequency	Composition ratio (%)
Nutritional management procedures	Yes	1087	69.63
	No	296	18.96
	Unclear	178	11.4
Nutritional management systems	Yes	1046	67.01
	No	332	21.27
	Unclear	183	11.72
Training plans covering both theoretical knowledge and practical skills	Yes	1081	69.25
	No	305	19.54
	Unclear	175	11.21
Emergency plans of nutritional management	Yes	934	59.83
	No	400	25.62
	Unclear	227	14.54
Multidisciplinary nutrition support teams	Yes	1262	80.85
	No	140	8.97
	Unclear	159	10.19
Monitoring the quality of nutritional management	Never	77	4.93
	Occasionally	297	19.03
	Sometimes	297	19.03
	Often	441	28.25
	Always	449	28.76
Refer patients to community nurses	Yes	1089	69.76
	No	472	30.24
Nutritional risk screening	Yes	1495	95.77
	No	66	4.23
Who conducts nutritional risk screening?	Doctors	280	17.94
	Nurses	392	25.11
	Dieticians	74	4.74
	Doctors and nurses	788	50.48
	Others	27	1.73
Developed related protocols to address potential complications	Yes	1383	88.6
	No	178	11.4
Regular follow-ups or track patients’ nutritional status after discharge	Yes	905	57.98
	No	656	42.02

### 3.4. Factors influencing nutritional management of chronic diseases among clinical nurses

Statistical analysis revealed several variables significantly associated with clinical nurses’ self-assessed knowledge, beliefs, and implementation of nutritional support for patients with chronic diseases.

Regarding self-assessed knowledge, significant differences were observed across variables such as age, years of work experience, professional title, clinical role, status as a nutrition support specialist nurse, and whether the department had established nutritional management processes, systems, training plans, emergency protocols, or community-based collaboration mechanisms. In contrast, variables such as educational background, the presence of a multidisciplinary nutrition support team, the frequency of quality analyses, implementation of nutritional risk screening, and screening methods showed no significant influence (see Supplementary Table S3).

Concerning beliefs, significant associations were identified with age, status as a nutrition support specialist nurse, and the presence of structural support elements such as established management processes and systems, training plans, emergency protocols, multidisciplinary teams, regular assessments with improvement strategies, coordination with community institutions, and standardized procedures for screening, complication management, and post-discharge follow-up. However, education level, years of experience, professional title, and clinical role were not significantly associated with beliefs (see Supplementary Table S4).

In terms of implementation, variables such as the presence of nutrition support specialists, institutional support structures (e.g., management protocols, systems, training plans, emergency plans), multidisciplinary teams, integration with community hospitals, and standardized procedures for complication management and follow-up showed significant associations. Conversely, demographic variables including age, sex, education, work experience, professional title, and role were not associated with differences in implementation behavior (see Supplementary Table S5).

### 3.5. Regression analysis

Because nutritional support self-assessed knowledge was treated as a binary variable, binary logistic regression analysis was conducted using understanding of nutritional support as the dependent variable (coded as 1 for “understanding” and 0 otherwise). Variables identified as significant in univariate analyses were included in multivariable modeling.

Binary logistic regression analysis revealed that role, nutrition support specialist nurse status, and the presence of a nutritional management system were significantly associated with nurses’self-assessed knowledge of nutritional support. Specifically, clinical nurses reported lower self-assessed knowledge compared with nursing administrators (odds ratio [OR] = 0.540, *P* = .030). Non-specialist nurses in nutrition support were more likely to report stronger self-assessed knowledge than their specialist counterparts (OR = 1.846, *P* < .001). Regarding nutritional management systems, nurses in departments without an established system reported higher self-assessed knowledge compared with those in departments with such a system (OR = 1.920, *P* = .028), while no significant difference was observed between the “unclear” group and the reference group (OR = 0.806, *P* = .459).

In contrast, variables such as age, years of work experience, professional title, clinical role, and the existence of nutritional management procedures or training plans did not show significant associations with self-assessed knowledge levels in the multivariate analysis (Table 4).

**Table 4:** Logistic regression analysis of knowledge of nutritional support.

Variable	β	Standard error	Wald	df	*P*	OR	95% CI	
							Lower	Upper
Constant	−0.177	0.392	0.204	1	.651	0.838		
Role								
Nursing administrator	-	-	-	-	-	1.000	-	-
Clinical nurse	−0.616	0.284	4.705	1	.030	0.540	0.309	0.942
Nutrition support specialist nurses or not?								
Yes	-	-	-	-	-	1.000	-	-
No	0.613	0.152	16.171	1	.000	1.846	1.369	2.488
Nutritional management systems			6.610	2	.037			
Yes	-	-	-	-	-	1.000	-	-
No	0.652	0.297	4.830	1	.028	1.920	1.073	3.435
Unclear	−0.216	0.291	0.549	1	.459	0.806	0.455	1.426

Because the total score for beliefs related to nutritional support was a continuous variable, linear regression analysis was employed. The total score on the Beliefs about Nutritional Support scale served as the dependent variable, and all variables found to be significant in univariate analysis were included in the model for further exploration.

The regression results indicated that regular analysis of nutrition management quality, the development of emergency response plans for adverse events related to chronic disease nutrition care, and routine follow-up or monitoring of patients’ nutritional status after discharge were all positively associated with stronger beliefs about nutritional support (*P* < .001). These findings suggest that structured quality improvement efforts and continuity of care can meaningfully reinforce nurses’ confidence in the value of nutritional support.

In contrast, variables such as age, nutrition support specialist status, the presence of chronic disease nutrition management protocols, and related training programs did not show significant associations with belief scores (*P* > .05). While these factors may contribute to broader institutional readiness, their direct influence on individual belief formation appears limited (Table 5).

**Table 5:** Linear regression analysis of nutritional support-related beliefs.

	Unstandardized coefficients		Standardized coefficient	t	*P*	95% CI for β		Collinearity statistics	
	β	Standard error	β			Lower bound	Upper bound	Tolerance	VIF
Constant	46.886	1.221		38.414	.000	44.491	49.280		
Emergency plans for nutritional management	−0.434	0.206	−0.068	−2.110	.035	−0.837	−0.030	0.456	2.192
Monitoring nutritional management	0.923	0.100	0.240	9.242	.000	0.727	1.119	0.695	1.439
Developed related protocols to address potential complications	−1.452	0.337	−0.098	−4.303	.000	−2.114	−0.790	0.902	1.109
Regular post-discharge follow-up/tracking of nutritional status	−1.559	0.227	−0.163	−6.870	.000	−2.004	−1.114	0.827	1.209

Because the total score for implementation of nutritional support was a continuous variable, linear regression analysis was conducted using the implementation score as the dependent variable. Variables identified as significant in univariate analysis were included in the model for further exploration.

Assessment of nutritional management, the establishment of protocols for managing enteral nutrition complications (including gastric retention, nausea and vomiting, feeding tube blockage, and aspiration), and routine follow-up or monitoring of nutritional status after discharge were all significantly associated with improved implementation (*P* < .001). These findings underscore the importance of structured clinical processes and continuity of care in enhancing the delivery of nutritional support.

In contrast, factors such as designation as a specialized nutrition support nurse, the extent of referral collaboration with community hospitals, and the development of chronic disease-related management protocols, policies, and training programs did not show significant associations with implementation outcomes. While these elements may contribute to broader institutional readiness, their direct influence on day-to-day implementation appears limited (Table 6).

**Table 6:** Linear regression analysis of the implementation of nutritional support.

	Unstandardized coefficients		Standardized coefficient	t	*P*	95% CI for β		Collinearity statistics	
	β	Standard error	β			Lower bound	Upper bound	Tolerance	VIF
Constant	75.247	2.835		26.538	0.000	69.686	80.809		
Assessment of nutritional management	2.766	0.240	0.286	11.538	0.000	2.296	3.236	0.696	1.436
Developed related protocols to address potential complications	−6.304	0.811	−0.170	−7.777	0.000	−7.894	−4.714	0.902	1.108
Regular post-discharge follow-up/tracking of nutritional status	−5.834	0.545	−0.244	−10.708	0.000	−6.903	−4.765	0.828	1.208

## 4. Discussion

This study on nutritional support for patients with chronic diseases revealed that most hospital departments have implemented foundational structures, including management protocols and systems, multidisciplinary teams, and training programs. However, gaps remain, particularly in post-discharge follow-up of patients’ nutritional status, which continues to be a weak link in continuity of care.

The sample included 40 hospitals across multiple regions and institutional tiers, with a predominance of tertiary grade A hospitals (70.00%) and teaching institutions (87.50%), many of which were located in economically developed areas such as Hangzhou. These characteristics may confer advantages in staffing, education, and clinical research, potentially influencing the higher levels of nutritional self-assessed knowledge, attitudes, and practices observed among participating nurses. Among the 1561 respondents, the majority were aged 20 to 40 years (80.72%), held a bachelor’s degree or higher (87.64%), and had 11 to 20 years of professional experience (33.63%). Most held mid-level professional titles (75.98% were nurses or senior nurses) and were working as clinical nurses (92.12%). Notably, only 13.97% were specialist nurses in nutritional support, suggesting that professional capacity in this domain remains limited and warrants strategic development.

More than 95% of departments conducted nutritional risk screening, and nearly 89% had complication management protocols in place. These data point to broad institutional support for nutritional care. However, only 57.98% of departments conducted regular follow-up or tracking after patient discharge, highlighting a key shortfall in comprehensive longitudinal nutritional management. In line with the European Society for Parenteral and Enteral Nutrition(ESPEN) guidelines on DRM, which emphasize early screening, structured follow-up, and nurse training, our study found that while many departments have established nutritional management protocols, post-discharge follow-up was not universally implemented. ESPEN guidelines recommend regular nutritional assessments for patients after discharge to prevent malnutrition and associated complications,^[16]^ yet our findings indicate a significant gap in this area. Addressing this discrepancy will be essential in improving DRM management and aligning with international best practices.

From the perspective of influencing factors, knowledge of nutritional support was associated with age, professional title, specialist status, and whether a nutrition management system had been established. Older nurses and those with higher professional ranks reported greater understanding, which may reflect accumulated clinical experience and broader professional exposure. Interestingly, specialist status was inversely associated with greater knowledge, warranting further investigation. Years of work experience alone did not significantly affect knowledge levels, suggesting that experience without targeted engagement in nutritional care may be insufficient. The negative predictors identified in our regression analysis, such as inadequate follow-up and limited nurse training, align with findings from other studies that have reported similar barriers to effective nutritional support in healthcare settings.^[17]^ These results may reflect resource limitations, insufficient standardized protocols, and gaps in interdisciplinary collaboration. Further investigation is needed to explore how these factors influence the outcomes of nurse-led nutrition interventions and to identify strategies for overcoming these challenges.

Our finding that specialized nutrition support nurses did not report superior self-assessed knowledge resonates with international reports highlighting variability in specialist training and role implementation. Conversely, the strong association between structured protocols and better practice aligns with core recommendations from ESPEN guidelines, emphasizing system-level enablers. The identified gap in post-discharge follow-up mirrors challenges documented in multi-country studies in Southeast Asia, suggesting common systemic barriers beyond regional context.

Beliefs about nutritional support were strongly influenced by structural and procedural elements, including regular assessments, availability of emergency response plans, and post-discharge follow-up practices, underscoring the value of embedded clinical processes in fostering positive attitudes. Similarly, these factors, along with standardized protocols for managing enteral nutrition complications, played a critical role in shaping implementation behaviors.

A noteworthy counterintuitive finding emerged from the regression analysis: the presence of emergency response plans, complication management protocols, and post-discharge follow-up practices was significantly associated with lower belief scores among nurses. At first glance, this appears contrary to expectation, as structured protocols are generally considered supportive of clinical practice and professional confidence. However, several possible explanations may account for this finding. First, reverse causality cannot be ruled out: departments that established these protocols may have been those with more pronounced nutritional challenges and lower baseline beliefs among nurses, with protocols implemented as a response to clinical needs rather than as drivers of belief formation. Second, the presence of protocols does not guarantee effective implementation: written policies without adequate training, monitoring, or feedback may fail to translate into positive beliefs and could even be perceived as burdensome, undermining professional motivation. Third, social desirability effects may play a role: in departments with well-established protocols, nurses may apply more stringent self-assessment standards when rating their beliefs, paradoxically reporting lower scores that reflect higher professional self-expectation rather than genuine lack of conviction. Fourth, there is a level-of-measurement distinction: our study assessed individual-level beliefs, whereas protocols represent organizational-level structural variables; unmeasured factors such as workload, department culture, and leadership support may moderate this relationship. Given the cross-sectional design of this study, causal inferences cannot be drawn, and these interpretations should be considered speculative pending validation through longitudinal or qualitative research.

Overall, while strong progress has been made in building the structural foundation for the nutritional management of chronic diseases, efforts to improve continuity of care and institutionalize follow-up processes remain essential. Targeted investment in training, staffing, and operational procedures will be key to strengthening nutritional care delivery and optimizing patient outcomes.

In the practical implementation of nutritional support, nurses’ knowledge and attitudes play a pivotal role in both information sharing and the overall effectiveness of care. Higher levels of nutritional knowledge and positive attitudes among nurses are closely associated with more frequent and effective communication regarding nutritional status, underscoring the importance of enhancing nurses’ competencies and confidence in nutritional care.^[18]^ A key barrier to effective nutritional support in hospitals remains the lack of standardized practices and adequate resources. Addressing this challenge requires stronger interdisciplinary collaboration, particularly between nurses and physicians, as well as improved organizational infrastructure.^[19]^ The role of nursing staff is central to the success of nutritional interventions. Hospitals with greater medical resources, robust staffing models, and strong teaching and research capabilities tend to report higher levels of nutritional knowledge, beliefs, and implementation among nursing staff. Increasing the number of professional nurses and support personnel has been linked to improved patient nutritional status and overall care quality.^[20]^ Additionally, redefining the nursing role in nutritional care, such as protecting mealtimes and adjusting work schedules, can enhance patients’ nutrient intake and satisfaction.^[21]^

This study had several limitations. First, although the sample included 40 hospitals, it was heavily weighted toward tertiary grade A institutions, potentially introducing sample bias and limiting generalizability to primary care settings. Second, the cross-sectional design does limit our ability to draw causal inferences, and this methodology does not capture the temporal changes in nutritional outcomes. Third, reliance on self-reported data introduces the possibility of social desirability and recall bias, which may affect data accuracy. Fourth, important contextual factors such as hospital culture and nursing workload were not fully explored, which may have constrained the comprehensiveness of the findings. Finally, although the measurement tools were validated in preliminary studies, the potential for measurement error remains. Knowledge was measured using a single self-report item that captured perceived confidence rather than objectively verified knowledge competency. This approach may be subject to social desirability bias and individual differences in self-assessment accuracy, and it does not allow for validation of construct validity. Future studies should employ validated, multi-dimensional knowledge instruments to objectively assess nurses’nutritional knowledge.

Future research should address these limitations by employing longitudinal or mixed-methods designs, expanding sampling to include more diverse healthcare settings and incorporating objective performance metrics. Exploring institutional culture and workload dynamics may also yield deeper insights into the barriers and facilitators of effective nutritional support. Furthermore, a residual risk of Type I error remains in our study. Findings near the conventional significance threshold should therefore be interpreted with caution.

## 5. Conclusions

In conclusion, we surveyed 1561 nurses across 40 hospitals and found that most departments had established chronic disease nutrition management protocols, multidisciplinary teams, and training programs. However, gaps remain, particularly in post-discharge follow-up and continuity of care. Factors influencing nurses’ self-assessed knowledge, beliefs, and implementation include age, professional title, specialist status, and the presence of institutional protocols. These findings provide a foundation for targeted interventions to strengthen nursing engagement and effectiveness in the nutritional management of patients with chronic disease.

## Author contributions

**Conceptualization:** Shuyan Li, Aixia Wang.

**Data curation:** Xiaoxia Huang, Huadi Yuan, Haiyan Zhou, Qiaoling Zhang, Yuehua Dai, Jiaying Tang, Xiuqin Feng.

**Investigation:** Shuyan Li, Aixia Wang, Xiaoxia Huang, Haiyan Zhou, Qiaoling Zhang, Yuehua Dai, Jiaying Tang, Xiuqin Feng.

**Methodology:** Huadi Yuan, Haiyan Zhou, Qiaoling Zhang, Yuehua Dai, Jiaying Tang, Xiuqin Feng.

**Supervision:** Shuyan Li, Aixia Wang, Xiaoxia Huang, Huadi Yuan, Haiyan Zhou, Qiaoling Zhang, Yuehua Dai, Jiaying Tang, Xiuqin Feng.

**Writing – review & editing:** Shuyan Li, Aixia Wang, Xiaoxia Huang.










