# Supplementary material for: Clinical nurses’self-assessed knowledge, beliefs, and practice in nutritional management of chronic disease patients: A cross-sectional survey in Zhejiang Province
Source: Medicine (Baltimore). 2026 Jun 5;105(23):e49154. doi: 10.1097/MD.0000000000049154 (PMC13246058; doi:10.1097/MD.0000000000049154)
Supplement: Supplementary file 1 [file medi-105-e49154-s001.docx]

**Supplementary Table 1. Nutrition support belief questionnaire**

|  | Strongly disagree | Disagree | Neutral | Agree | Strongly agree |
| --- | --- | --- | --- | --- | --- |
| 1. How important do you think it is to have knowledge and skills related to nutritional care for chronic diseases? | | | | | |
|  | 0 (0.00) | 4 (0.26) | 85 (5.45) | 375 (24.02) | 1097 (70.28) |
| 2. What is your level of satisfaction with your own knowledge and skills related to chronic disease nutrition care? | | | | | |
|  | 10 (0.64) | 124 (7.94) | 714 (45.74) | 336 (21.52) | 377 (24.15) |
| 3. How confident are you in applying your nutrition care knowledge and skills to meet clinical practice needs? | | | | | |
|  | 11 (0.70) | 78 (5.00) | 705 (45.16) | 517 (33.12) | 250 (16.02) |
| 4. What is your opinion on the importance of conducting nutrition risk screening and assessment for patients with chronic diseases? | | | | | |
|  | 0 (0.00) | 2 (0.13) | 126 (8.07) | 405 (25.94) | 1028 (65.86) |
| 5. Rank your agreement: attention should be paid to the nutritional support of patients with chronic diseases in your work. | | | | | |
|  | 3 (0.19) | 1 (0.06) | 70 (4.48) | 362 (23.19) | 1125 (72.07) |
|  | Strongly disagree | Disagree | Neutral | Agree | Strongly agree |
| 6. How important do you think nutritional support is for the treatment and recovery of patients with chronic diseases? | | | | | |
|  | 3 (0.19) | 0 (0.00) | 53 (3.40) | 337 (21.59) | 1168 (74.82) |
| 7. Do you believe that education and guidance on nutrition support should be provided to patients with chronic diseases and their families? | | | | | |
|  | 5 (0.32) | 0 (0.00) | 47 (3.01) | 338 (21.65) | 1171 (24.98) |
| 8. Do you think nutritional support care should be provided for patients with chronic diseases? | | | | | |
|  | 3 (0.19) | 0 (0.00) | 50 (3.20) | 334 (21.40) | 1174 (75.21) |
| 9. Do you believe that the intake and output, as well as complications, of chronic disease patients receiving enteral and parenteral nutrition support therapy should be closely monitored? | | | | | |
|  | 4 (0.26) | 2 (0.13) | 45 (2.88) | 353 (22.61) | 1157 (74.12) |
| 10. Do you proactively seek to learn about enteral and parenteral nutrition? | | | | | |
|  | 31 (1.99) | 325 (20.82) | 495 (31.71) | 398 (25.50) | 312 (19.99) |
