# Supplementary material for: Clinical nurses’self-assessed knowledge, beliefs, and practice in nutritional management of chronic disease patients: A cross-sectional survey in Zhejiang Province
Source: Medicine (Baltimore). 2026 Jun 5;105(23):e49154. doi: 10.1097/MD.0000000000049154 (PMC13246058; doi:10.1097/MD.0000000000049154)
Supplement: Supplementary file 2 [file medi-105-e49154-s002.docx]

**Supplementary Table 2. Survey of implementation of nutritional support**

|  | Always | Frequently | Sometimes | Occasionally | Never |
| --- | --- | --- | --- | --- | --- |
| 1. Before and after each feeding, you flush the tube with warm water using a pulsed technique. | | | | | |
|  | 1281 (82.06) | 204 (13.07) | 37 (2.37) | 26 (1.67) | 13 (0.83) |
| 2. You administer liquid medications at pH ≤ 5 separately from nutrition solutions. | | | | | |
|  | 934 (59.83) | 253 (16.21) | 163 (10.44) | 133 (8.52) | 78 (5.00) |
| 3. When administering parenteral nutrition, you give priority to intermittent infusion for patients with peripheral venous access. | | | | | |
|  | 291 (18.64) | 198 (12.68) | 294 (18.83) | 350 (22.42) | 428 (27.42) |
| 4. When administering parenteral nutrition, you consider cyclic infusion for patients who require parenteral nutrition for more than 2 weeks. | | | | | |
|  | 352 (22.55) | 323 (20.69) | 406 (26.01) | 314 (20.12) | 166 (10.63) |
| 5. When administering parenteral nutrition, the infusion rate is determined based on the patient’s nutritional needs and treatment situation; for intermittent infusions, the rate is 200–300 mL/h. | | | | | |
|  | 475 (30.43) | 355 (22.74) | 328 (21.01) | 216 (13.84) | 187 (11.98) |
|  | Always | Frequently | Sometimes | Occasionally | Never |
| 6. When administering parenteral nutrition infusion, you replace the infusion set at least once every 24 h. | | | | | |
|  | 1312 (84.05) | 139 (8.90) | 54 (3.46) | 48 (3.07) | 8 (0.51) |
| 7. All nutrient solutions in your department are prepared freshly as needed, with clear labeling applied after preparation. Nutrient solutions that require storage are kept refrigerated and protected from light. | | | | | |
|  | 1176 (75.34) | 183 (11.72) | 74 (4.74) | 66 (4.23) | 62 (3.97) |
| 8. You select appropriate enteral nutrition formulas based on the patient’s gastrointestinal function. | | | | | |
|  | 1073 (68.74) | 259 (16.59) | 107 (6.85) | 88 (5.64) | 34 (2.18) |
| 9. You complete the infusion of parenteral nutrition solution within 24 h, and the lipid emulsion is infused for less than 12 h or according to the instructions in the medication leaflet. | | | | | |
|  | 1222 (78.28) | 212 (13.58) | 70 (4.48) | 47 (3.01) | 10 (0.64) |
| 10. You are able to identify and manage complications of parenteral nutrition (such as phlebitis, infections, catheter thrombosis, abnormal blood glucose, lipid emulsion allergy, refeeding syndrome, etc.). | | | | | |
|  | 806 (51.63) | 393 (25.18) | 232 (14.86) | 110 (7.05) | 20 (1.28) |
|  | Always | Frequently | Sometimes | Occasionally | Never |
| 11. For patients with long-term nasal feeding tubes, you replace the tube to the other nostril every 4–6 weeks. | | | | | |
|  | 1040 (66.62) | 253 (16.21) | 129 (8.26) | 82 (5.25) | 57 (3.65) |
| 12. Before the procedure, you assess the patient’s blood vessels, infusion fluids, and infusion devices. | | | | | |
|  | 1176 (75.34) | 246 (15.76) | 83 (5.32) | 42 (2.69) | 14 (0.90) |
| 13. You make appropriate choices regarding the infusion method based on the duration of treatment, the osmolarity of the nutrient solution, and the specific condition of the patient. | | | | | |
|  | 931 (59.64) | 330 (21.14) | 172 (11.02) | 94 (6.02) | 34 (2.18) |
| 14. You are able to identify the nutritional and health education needs of patients and caregivers. | | | | | |
|  | 568 (36.39) | 403 (25.82) | 357 (22.87) | 204 (13.07) | 29 (1.86) |
| 15. You are able to identify the factors that facilitate or hinder patients and caregivers from accepting nutrition and health education. | | | | | |
|  | 551 (35.30) | 404 (25.88) | 386 (24.73) | 188 (12.04) | 32 (2.05) |
|  | Always | Frequently | Sometimes | Occasionally | Never |
| 16. You adjust nutrition and health education according to the different stages of a patient’s hospitalization. | | | | | |
|  | 579 (37.09) | 435 (27.87) | 342 (21.91) | 172 (11.02) | 33 (2.11) |
| 17. You provide patients with personalized nutrition education and guidance. | | | | | |
|  | 566 (36.26) | 456 (29.21) | 329 (21.08) | 177 (11.34) | 33 (2.11) |
| 18. You adjust your health education plans in a timely manner based on the evaluations of nutrition and health education? | | | | | |
|  | 573 (36.71) | 442 (28.32) | 336 (21.52) | 177 (11.34) | 33 (2.11) |

PH=potential of Hydrogen
