# Supplementary material for: Clinical nurses’self-assessed knowledge, beliefs, and practice in nutritional management of chronic disease patients: A cross-sectional survey in Zhejiang Province
Source: Medicine (Baltimore). 2026 Jun 5;105(23):e49154. doi: 10.1097/MD.0000000000049154 (PMC13246058; doi:10.1097/MD.0000000000049154)
Supplement: Supplementary file 3 [file medi-105-e49154-s003.docx]

**Supplementary Table 3. Univariate analysis of clinical nurses’ knowledge of nutritional support for patients with chronic disease**

| **Variable** | **Category** | **Not knowledgeable** | **knowledgeable** | **χ^2^** | **P** |
| --- | --- | --- | --- | --- | --- |
| Age (years) | 20–30 | 306 (48.7) | 322 (51.3) | 33.759 | **<0.001** |
|  | 31–40 | 256 (40.5) | 376 (59.5) |  |  |
|  | 41–50 | 81 (30.5) | 185 (69.5) |  |  |
|  | ≥51 | 7 (20.0) | 28 (80.0) |  |  |
| Education | Senior High School/Vocational High School | 1 (25.0) | 3 (75.0) | 3.550 | 0.314 |
|  | Junior College | 84 (43.5) | 109 (56.5) |  |  |
|  | Bachelor’s Degree | 556 (41.8) | 775 (58.2) |  |  |
|  | Master’s Degree | 9 (27.3) | 24 (72.7) |  |  |
| Work experience  (years) | <1 | 43 (56.6) | 33 (43.4) | 34.546 | **<0.001** |
|  | 1–3 | 96 (44.4) | 120 (55.6) |  |  |
|  | 4–6 | 104 (48.4) | 111 (51.6) |  |  |
|  | 7–10 | 144 (47.8) | 157 (52.2) |  |  |
|  | 11–20 | 196 (37.3) | 329 (62.7) |  |  |
|  | >20 | 67 (29.4) | 161 (70.6) |  |  |
| Job titles | Nurse | 118 (51.3) | 112 (48.7) | 42.438 | **<0.001** |
|  | Registered Nurse | 287 (46.9) | 325 (53.1) |  |  |
|  | Nurse Supervisor | 212 (36.9) | 362 (63.1) |  |  |
|  | Deputy Chief Nurse | 29 (23.4) | 95 (76.6) |  |  |
|  | Chief Nurse | 4 (19.0) | 17 (81.0) |  |  |
| Role | Nursing Administrator | 24 (19.5) | 99 (80.5) | 26.903 | **<0.001** |
|  | Clinical Nurse | 626 (43.5) | 812 (56.5) |  |  |
| Nutrition support specialist nurse? | Yes | 121 (55.5) | 97 (44.5) | 20.043 | **<0.001** |
|  | No | 529 (39.4) | 814 (60.6) |  |  |
| Nutritional management procedures | Yes | 477 (43.9) | 610 (56.1) | 13.695 | **0.001** |
|  | No | 95 (32.1) | 201 (67.9) |  |  |
|  | Unclear | 78 (43.8) | 100 (56.2) |  |  |
| Nutritional management systems | Yes | 466 (44.6) | 580 (55.4) | 24.477 | **<0.001** |
|  | No | 99 (29.8) | 233 (70.2) |  |  |
|  | Unclear | 85 (46.4) | 98 (53.6) |  |  |
| Training plans covering both theoretical knowledge and practical skills | Yes | 469 (43.4) | 612 (56.6) | 11.673 | **0.003** |
|  | No | 101 (33.1) | 204 (66.9) |  |  |
|  | Unclear | 80 (45.7) | 95 (54.3) |  |  |
| Emergency plans of nutritional management | Yes | 421 (45.1) | 513 (54.9) | 18.649 | **<0.001** |
|  | No | 130 (32.5) | 270 (67.5) |  |  |
|  | Unclear | 99 (43.6) | 128 (56.4) |  |  |
| Multidisciplinary nutrition support teams | Yes | 523 (41.1) | 739 (58.6) | 1.757 | 0.415 |
|  | No | 54 (38.6) | 86 (61.4) |  |  |
|  | Unclear | 73 (45.9) | 86 (54.1) |  |  |
| Monitoring the quality of nutritional management | Never | 34 (44.2) | 43 (55.8) | 3.863 | 0.425 |
|  | Occasionally | 125 (42.1) | 172 (57.9) |  |  |
|  | Sometimes | 109 (36.7) | 188 (63.3) |  |  |
|  | Often | 191 (43.3) | 250 (56.7) |  |  |
|  | Always | 191 (42.5) | 258 (57.5) |  |  |
| Refer patients to community nurses | Yes | 486 (44.6) | 603 (55.4) | 13.233 | **<0.001** |
|  | No | 164 (34.7) | 308 (65.3) |  |  |
| Regular post-discharge follow-up/tracking of patient nutritional status | Yes | 407 (45.0) | 498 (55.0) | 9.841 | **0.002** |
|  | No | 243 (37.0) | 413 (63.0) |  |  |
