# Supplementary material for: Clinical nurses’self-assessed knowledge, beliefs, and practice in nutritional management of chronic disease patients: A cross-sectional survey in Zhejiang Province
Source: Medicine (Baltimore). 2026 Jun 5;105(23):e49154. doi: 10.1097/MD.0000000000049154 (PMC13246058; doi:10.1097/MD.0000000000049154)
Supplement: Supplementary file 4 [file medi-105-e49154-s004.docx]

**Supplementary Table 4. Univariate analysis of clinical nurses’ beliefs regarding nutritional support for patients with chronic disease**

| **Variable** | **Category** | **M (P_25_, P_75_)** | ***Z*** | **P** |
| --- | --- | --- | --- | --- |
| Age (years) | 20–30 | 44.0 (41.0, 47.0) | 8.115 | 0.044 |
|  | 31–40 | 44.0 (40.3, 47.0) |  |  |
|  | 41–50 | 44.0 (40.8, 46.0) |  |  |
|  | ≥51 | 41.0 (37.0, 46.0) |  |  |
| Education | Senior High School/Vocational High School | 45.0 (38.0, 49.0) | 0.313 | 0.958 |
|  | Junior College | 44.0 (40.0, 47.0) |  |  |
|  | Bachelor’s Degree | 44.0 (40.0, 47.0) |  |  |
|  | Master’s Degree | 44.0 (41.5, 46.0) |  |  |
| Work experience  (years) | <1 | 45.0 (41.3, 47.0) | 5.412 | 0.368 |
|  | 1–3 | 45.0 (41.0, 47.0) |  |  |
|  | 4–6 | 44.0 (41.0, 46.0) |  |  |
|  | 7–10 | 44.0 (40.0, 47.0) |  |  |
|  | 11–20 | 44.0 (40.5, 47.0) |  |  |
|  | >20 | 44.0 (40.0, 46.0) |  |  |
| Job titles | Nurse | 45.0 (40.8, 48.0) | 5.734 | 0.220 |
|  | Registered Nurse | 44.0 (41.0, 47.0) |  |  |
|  | Nurse Supervisor | 44.0 (40.0, 47.0) |  |  |
|  | Deputy Chief Nurse | 44.0 (41.0, 46.0) |  |  |
|  | Chief Nurse | 44.0 (41.5, 47.0) |  |  |
| Role | Nursing Administrator | 44.0 (41.0, 46.0) | 0.769 | 0.442 |
|  | Clinical Nurse | 44.0 (40.0, 47.0) |  |  |
| Nutrition support specialist nurse? | Yes | 45.0 (40.0, 48.0) | 2.483 | **0.013** |
|  | No | 44.0 (41.0, 47.0) |  |  |
| Nutritional management procedures | Yes | 45.0 (42.0, 48.0) | 224.694 | **<0.001** |
|  | No | 42.0 (38.0, 44.0) |  |  |
|  | Unclear | 41.0 (37.0, 44.0) |  |  |
| Nutritional management systems | Yes | 45.0 (43.0, 48.0) | 221.214 | **<0.001** |
|  | No | 42.0 (38.0, 44.0) |  |  |
|  | Unclear | 41.0 (36.0, 44.0) |  |  |
| Training plans covering both theoretical knowledge and practical skills | Yes | 45.0 (42.0, 48.0) | 228.155 | **<0.001** |
|  | No | 42.0 (38.0, 44.0) |  |  |
|  | Unclear | 41.0 (36.0, 44.0) |  |  |
| Emergency plans of nutritional management | Yes | 45.0 (43.0, 48.0) | 250.186 | **<0.001** |
|  | No | 42.0 (38.0, 44.0) |  |  |
|  | Unclear | 42.0 (37.0, 44.0) |  |  |
| Multidisciplinary nutrition support teams | Yes | 45.0 (41.0, 47.0) | 130.325 | **<0.001** |
|  | No | 42.0 (37.0, 44.0) |  |  |
|  | Unclear | 41.0 (37.0, 44.0) |  |  |
| Monitoring the quality of nutritional management | Never | 42.0 (37.0, 45.0) | 350.563 | **<0.001** |
|  | Occasionally | 41.0 (37.0, 44.0) |  |  |
|  | Sometimes | 43.0 (39.0, 45.0) |  |  |
|  | Often | 44.0 (40.5, 46.0) |  |  |
|  | Always | 47.0 (44.0, 49.0) |  |  |
| Refer patients to community nurses  Nutritional risk screening | Yes | 45.0 (42.0, 48.0) | 12.113 | **<0.001** |
|  | No | 42.0 (38.0, 44.0) |  |  |
| Nutritional risk screening | Yes | 44.0 (41.0, 47.0) | 6.648 | **<0.001** |
|  | No | 40.5 (35.0, 43.0) |  |  |
| Who conducts nutritional risk screening? | Doctors | 44.0 (41.0, 46.0) | 31.750 | **<0.001** |
|  | Nurses | 44.0 (40.0, 46.0) |  |  |
|  | Dieticians | 44.0 (40.8, 47.0) |  |  |
|  | Doctors and nurses | 45.0 (41.0, 47.0) |  |  |
|  | Others | 40.0 (36.0, 43.0) |  |  |
| Developed related protocols to address potential complications | Yes | 44.0 (41.0, 47.0) | 8.922 | **<0.001** |
|  | No | 41.0 (36.8, 44.0) |  |  |
| Regular post-discharge follow-up or tracking of patient nutritional status | Yes | 45.0 (42.0, 48.0) | 13.738 | **<0.001** |
|  | No | 43.0 (38.0, 45.0) |  |  |
