# Supplementary material for: Clinical nurses’self-assessed knowledge, beliefs, and practice in nutritional management of chronic disease patients: A cross-sectional survey in Zhejiang Province
Source: Medicine (Baltimore). 2026 Jun 5;105(23):e49154. doi: 10.1097/MD.0000000000049154 (PMC13246058; doi:10.1097/MD.0000000000049154)
Supplement: Supplementary file 5 [file medi-105-e49154-s005.docx]

**Supplementary Table 5. Univariate analysis of clinical nurses’ nutritional support behaviors for patients with chronic diseases**

| **Variable** | **Category** | **M (P_25_,P_75_)** | **χ^2^** | **P** |
| --- | --- | --- | --- | --- |
| Age (years) | 20–30 | 75.0 (67.0, 83.8) | 2.226 | 0.527 |
|  | 31–40 | 74.0 (66.0, 82.0) |  |  |
|  | 41–50 | 75.0 (67.0, 81.0) |  |  |
|  | ≥51 | 71.0 (67.0, 81.0) |  |  |
| Education | Senior High School/Vocational High School | 78.5 (71.8, 83.8) | 4.792 | 0.188 |
|  | Junior College | 73.0 (66.0, 83.0) |  |  |
|  | Bachelor’s Degree | 75.0 (67.0, 82.0) |  |  |
|  | Master’s Degree | 72.0 (59.0, 79.5) |  |  |
| Work experience  (years) | <1 | 72.0 (65.8, 80.8) | 8.467 | 0.132 |
|  | 1–3 | 75.0 (68.0, 85.0) |  |  |
|  | 4–6 | 74.0 (65.0, 82.0) |  |  |
|  | 7–10 | 76.0 (67.0, 84.0) |  |  |
|  | 11–20 | 74.0 (66.0, 81.5) |  |  |
|  | >20 | 75.0 (67.0, 82.0) |  |  |
| Job titles | Nurse | 74.0 (68.0, 85.0) | 5.176 | 0.270 |
|  | Registered Nurse | 75.0 (67.0, 83.0) |  |  |
|  | Nurse Supervisor | 74.0 (66.0, 82.0) |  |  |
|  | Deputy Chief Nurse | 74.0 (67.0, 81.0) |  |  |
|  | Chief Nurse | 78.0 (71.0, 84.5) |  |  |
| Role | Nursing Administrator | 75.0 (67.0, 81.0) | 0.248 | 0.804 |
|  | Clinical Nurse | 74.0 (67.0, 83.0) |  |  |
| Nutrition support specialist nurse? | Yes | 78.0 (67.0, 86.0) | 2.579 | **0.010** |
|  | No | 74.0 (67.0, 82.0) |  |  |
| Nutritional management procedures | Yes | 78.0 (70.0, 84.0) | 175.351 | **<0.001** |
|  | No | 69.0 (62.0, 75.0) |  |  |
|  | Unclear | 68.0 (56.0, 75.0) |  |  |
| Nutritional management systems | Yes | 78.0 (70.0, 85.0) | 179.317 | **<0.001** |
|  | No | 69.5 (62.3, 76.0) |  |  |
|  | Unclear | 68.0 (56.0, 76.0) |  |  |
| Training plans covering both theoretical knowledge and practical skills | Yes | 78.0 (70.0, 84.0) | 178.238 | **<0.001** |
|  | No | 70.0 (62.5, 75.0) |  |  |
|  | Unclear | 67.0 (55.0, 75.0) |  |  |
| Emergency plans of nutritional management | Yes | 78.0 (70.8, 85.0) | 202.654 | **<0.001** |
|  | No | 70.0 (63.0, 77.0) |  |  |
|  | Unclear | 68.0 (59.0, 76.0) |  |  |
| Multidisciplinary nutrition support teams | Yes | 76.0 (68.0, 83.0) | 82.383 | **<0.001** |
|  | No | 69.0 (62.0, 76.0) |  |  |
|  | Unclear | 68.0 (60.0, 76.0) |  |  |
| Monitoring the quality of nutritional management | Never | 67.0 (59.0, 74.0) | 363.045 | **<0.001** |
|  | Occasionally | 67.0 (58.5, 74.0) |  |  |
|  | Sometimes | 71.0 (64.0, 79.0) |  |  |
|  | Often | 74.0 (68.0, 80.0) |  |  |
|  | Always | 83.0 (76.5, 88.0) |  |  |
| Refer patients to community nurses | Yes | 77.0 (70.0, 84.0) | 11.680 | **<0.001** |
|  | No | 69.0 (62.0, 76.0) |  |  |
| Nutritional risk screening | Yes | 75.0 (67.0, 83.0) | 3.821 | **<0.001** |
|  | No | 69.0 (55.0, 76.3) |  |  |
| Who conducts nutritional risk screening? | Doctors | 72.5 (66.3, 82.0) | 31.690 | **<0.001** |
|  | Nurses | 73.0 (65.0, 80.0) |  |  |
|  | Dieticians | 73.0 (65.0, 83.0) |  |  |
|  | Doctors and nurses | 76.0 (68.0, 84.0) |  |  |
|  | Others | 68.0 (55.0, 77.0) |  |  |
| Developed related protocols to address potential complications | Yes | 76.0 (68.0, 83.0) | 11.670 | **<0.001** |
|  | No | 65.0 (56.0, 71.0) |  |  |
| Regular post-discharge follow-up or tracking of patient nutritional status | Yes | 80.0 (71.5, 86.0) | 16.625 | **<0.001** |
|  | No | 69.0 (62.0, 76.0) |  |  |
